# Surgical Site Infections in a Tertiary Hospital in Egypt: Bacterial Isolates and Antibiotic Sensitivity Profiles

**DOI:** 10.7759/cureus.91151

**Published:** 2025-08-27

**Authors:** Amgad A Ezzat Othman, Esraa R Abu Bakr, Mohamed s Hemeda mohamed, Mohamed Abdel Basit Ibrahim Mohamed, Gamal G. Shimy Mohammed, Ayman S Yassin Alsayed

**Affiliations:** 1 Department of Medical Microbiology and Immunology, Faculty of Medicine, Al-Azhar University, Assiut, EGY; 2 Department of Forensic Medicine and Clinical Toxicology, Port Said University, Port Said, EGY; 3 Department of General Surgery, Faculty of Medicine, Al-Azhar University, Assiut, EGY

**Keywords:** antimicrobial resistance, egypt, postoperative infection, staphylococcus aureus, surgical site infections, vitek-2

## Abstract

Background

Surgical Site Infections (SSIs) are among the most common and costly postoperative complications, significantly impacting patient recovery and healthcare systems. Their burden is especially pronounced in low- and middle-income countries due to limited resources and inconsistent infection control practices.

Objective

To investigate the prevalence, microbial aetiology, and antimicrobial resistance patterns of SSIs in Al-Azhar University Hospital, Assiut, Egypt.

Methods

A prospective surveillance study was conducted on 90 patients undergoing general, gynaecological, urological, orthopaedic, and neurosurgical procedures. Patients were monitored for up to 90 days postoperatively. Wound swabs were collected, cultured, and analysed using both conventional microbiological methods and the automated VITEK-2 system (bioMérieux, France). Antibiotic susceptibility was assessed via the Kirby-Bauer disk diffusion method (Thermo Fisher Scientific, Basingstoke, UK) and VITEK-2.

Results

The most frequently isolated pathogen was *Staphylococcus aureus* (31.1%), followed by *Escherichia coli* and coagulase-negative staphylococci (16.7% each). High resistance rates were observed for ampicillin and gentamicin. Notably, a strong concordance was found between disc diffusion and VITEK-2 results (e.g., κ = 1 for ciprofloxacin and ampicillin; p<0.001). Patients with diabetes exhibited significantly longer hospital stays and were more likely to be admitted to the vascular surgery department.

Conclusion

SSIs continue to pose a major challenge in surgical care at Al-Azhar University Hospitals. The dominance of multidrug-resistant pathogens underscores the urgent need for improved infection control protocols and the rational use of antibiotics tailored to local resistance profiles.

## Introduction

Surgical Site Infections (SSIs) remain a major challenge for health systems worldwide. They often lead to more pain for patients, longer hospital stays, and a greater risk of death after surgery. Roughly 2-5% of all surgical patients globally develop an SSI, and these infections now make up almost 20% of all infections linked to healthcare. According to the latest WHO and CDC reports (2019-2023), these infections account for an estimated 3-8 million disability-adjusted life years (DALYs) lost each year, underlining the toll they take on public health [1,2]. 

The pathogenesis of SSIs is multifactorial, influenced by intrinsic patient-related factors (e.g., diabetes mellitus, immunosuppression, obesity), procedural variables (e.g., wound class, duration of surgery), and environmental conditions within the operating theatre. Notably, the majority of SSI pathogens originate from the patient's endogenous microbiota; however, exogenous contamination from surgical instruments, operating room air, or healthcare personnel also plays a role [3,4]. 

Globally, *Staphylococcus aureus*, including methicillin-resistant strains (MRSA), remains the most commonly isolated organism. However, the emergence of multidrug-resistant Gram-negative bacilli such as *Escherichia coli *and *Klebsiella* spp. pose a growing challenge, especially in low-resource settings where antimicrobial stewardship programs are limited or inconsistently applied [5, 6]. 

In Egypt, data regarding the prevalence and microbial profiles of SSIs are limited, particularly in Upper Egypt and tertiary referral centres such as Al-Azhar University Hospitals. Local studies are essential to understand the regional patterns of infection, resistance trends, and to evaluate the effectiveness of existing infection prevention strategies.

This study was therefore designed as a prospective surveillance investigation to determine the incidence of SSIs, identify the causative organisms, and evaluate their antimicrobial susceptibility profiles among patients undergoing various surgical procedures at Al-Azhar University Hospitals in Assiut, Egypt. The results aim to inform evidence-based infection control policies and guide the rational use of antibiotics in clinical practice.

This investigation primarily sought to delineate the microbiological entities and their corresponding antimicrobial resistance profiles implicated in SSIs recorded at the Assiut branch of Al-Azhar University Hospitals. Secondary objectives comprised the quantification of agreement between traditional antibiograms and results generated by the automated VITEK-2 apparatus, as well as the examination of clinical variables including underlying patient comorbidities, the specific surgical discipline involved, and the duration of the hospital admission.

## Materials and methods

Study design

This prospective observational study was conducted from January 2024 to June 2024 at Al-Azhar University Hospitals (Assiut branch), a tertiary referral centre located in Upper Egypt. The study aimed to investigate the prevalence of SSIs, their microbial aetiologies, and antimicrobial resistance patterns among patients undergoing various surgical procedures. Ethical approval was obtained from the Institutional Review Board (IRB) of the Faculty of Medicine, Al-Azhar University (approval number MSC/AZ.AST. /MIC007/14/227/1/2024), and written informed consent was obtained from all participants.

Study population

Ninety patients were enrolled over the study period. Eligible subjects included individuals undergoing general surgery or participating in orthopaedic, obstetric-gynaecological, urologic, or neurosurgical procedures. The inclusion criteria required the emergence of signs of infection within 30 days of surgery, or within 90 days if the patient possessed an implanted medical device. Uncontrolled diabetes, prior chemotherapy or radiotherapy, and infection onset after the 90-day postoperative threshold were grounds for exclusion. The decision to omit subjects with uncontrolled diabetes stemmed from the condition’s association with impaired wound healing and its established role as an independent risk factor for postoperative infections. Retaining these individuals could obscure the effects of surgery and microbiological variables by introducing confounding triggers from systemic disease. Clinical assessments of SSIs were performed daily during the hospital stay and throughout the postoperative follow-up; identification adhered to CDC criteria that categorise infections into superficial incisional, deep incisional, or organ/space entities.

Sample collection and microbiological processing

Exudate samples from abscess cavities were obtained with sterile swabs following strict aseptic procedures, ideally at the first appearance of inflammation and before any empirical antibiotic therapy began. Specimens were immediately transferred to the microbiology laboratory in appropriate transport media. Each was inoculated onto a battery of growth media: nutrient agar, blood agar, MacConkey agar, mannitol salt agar, and Sabouraud dextrose agar, with incubation at 37°C for 24 to 48 hours, exposing cultures to both aerobic and anaerobic conditions. Bacterial growth was characterised by colony appearance, Gram stain results, and classical biochemical methods, including catalase, coagulase, indole, urease, triple sugar iron agar reactions, citrate utilisation, and oxidase tests. Fungal growth was screened by the germ tube test to detect the presence of *Candida albicans*. Since the study concentrated on bacterial pathogens, antifungal assessment was restricted to this economical and expedient technique that is practical in laboratories with limited resources; detailed identification of candida at the species level was not undertaken.

Antimicrobial susceptibility testing

The antibiotic sensitivity profile of each isolate was determined using two methods: the Kirby-Bauer disk diffusion method (Thermo Fisher Scientific, Basingstoke, UK) and the automated VITEK-2 system (bioMérieux, France). For disk diffusion, bacterial suspensions were adjusted to a 0.5 McFarland standard and inoculated on Mueller-Hinton agar plates. Discs containing commonly used antibiotics were applied, and plates were incubated at 35-37°C for 18-24 hours. Zone diameters were interpreted according to Clinical Laboratory Standards Institute (CLSI) guidelines [7]. In parallel, isolates were tested using the VITEK-2 system for confirmation and minimum inhibitory concentration (MIC) determination. Quality control was maintained using standard strains: *Escherichia coli* ATCC 25922 and *Pseudomonas aeruginosa* ATCC 27853.

Data management and statistical analysis

Clinical and laboratory parameters were systematically documented for every subject, encompassing age, sex, comorbidities, surgical type, wound classification, microbiological cultures, and length of hospital admission. Data management and statistical evaluation were conducted employing IBM SPSS Statistics for Windows, Version 25 (Released 2011; IBM Corp., Armonk, New York, United States). Descriptive statistics for continuous variables were conveyed as mean ± standard deviation (SD) or as median with interquartile range (IQR), while categorical variables were represented as absolute frequencies and corresponding percentages. Comparative analyses utilized the Chi-square test for categorical outcomes and applied the Mann-Whitney U test or analysis of variance (ANOVA), according to the distribution and number of groups in continuous variables. To ascertain determinants of SSIs, a logistic regression framework was employed. Independent variables incorporated in the regression encompassed age, sex, diabetes mellitus, hypertension, body mass index category, wound classification, surgical modality, and baseline laboratory parameters, specifically haemoglobin and total leukocyte count. Confounding variables were controlled by inclusion in the multivariable regression schematic, and only those with a p-value below 0.1 in univariable examination were retained for the conclusive model. A threshold of p<0.05 was uniformly applied to designate statistical significance.

## Results

Baseline characteristics

A total of 90 patients were included in the study, with a mean age of 42.3±19.2 years (range: 7-90 years). The majority were female patients (66.7%), while male patients accounted for 33.3% of the sample. Regarding body mass index (BMI), more than half of the participants were classified as obese (53.3%), followed by overweight (25.6%), and normal weight (21.1%). A quarter of the patients were smokers (24.4%). Concerning comorbidities, 51.1% of patients had diabetes mellitus, 35.6% had hypertension, and 11.1% had cardiac disease. Notably, 80% of the participants had more than one comorbidity (combined), as shown in Table 1.

**Table 1:** Baseline characteristics of the study population (n=90)

Variable	Category	Value
Age (years)	Mean ± SD	42.3 ± 19.2
	Range	7-90
Gender	Male	30 (33.3%)
	Female	60 (66.7%)
BMI (kg/m²)	Mean ± SD	29.2 ± 4.4
	Range	19-37
BMI Category	Normal weight	19 (21.1%)
	Overweight	23 (25.6%)
	Obese	48 (53.3%)
Smoking	Yes	22 (24.4%)
	No	68 (75.6%)
Comorbidities	Diabetes mellitus	46 (51.1%)
	Hypertension	32 (35.6%)
	Cardiac disease	10 (11.1%)
	Liver disease	2 (2.2%)
	Malignancy	0 (0%)
Comorbidity pattern	Single comorbidity	18 (20%)
	Combined comorbidities	72 (80%)

Preoperative characteristics and wound classification

Among the 90 patients, 72.2% received preoperative antibiotic prophylaxis, while 68.9% reported taking a preoperative shower. However, 45.6% had their body hair shaved with a razor before surgery, which may increase the risk of infection. Regarding wound classification, most surgical wounds were classified as clean or clean-contaminated (Class I & II), representing 76.7%, while the remaining 23.3% were classified as contaminated or dirty (Class III & IV) [3]. Notably, 81.8% of patients had a surgical drain placed postoperatively [3]. These findings are summarised in Table 2.

**Table 2:** Preoperative data and wound classification (n=90) [3]

Variable	Frequency (n)	Percentage (%)
Preoperative showering	62	68.9%
Preoperative shaving with a razor	41	45.6%
Preoperative antibiotic prophylaxis	65	72.2%
Wound classification		
– Class I & II (Clean/Clean-contaminated)	69	76.7%
– Class III & IV (Contaminated/Dirty)	21	23.3%
Presence of drain tube	73	81.8%

Surgical procedures and departmental distribution

The dataset reflected an equal distribution of cases for cholecystectomy, diabetic foot surgery, and a group of miscellaneous operative interventions, with each grouping contributing 13.3% of total surgical volume. Cesarean delivery and orthopaedic procedures each represented 11.1% of the cases. The next tier of interventions comprised appendectomy and total nephrectomy for renal cyst, each accounting for 8.9%; management of urinary calculi (7.8%); elective and emergency hernia repairs (6.7%); and perineal tear repairs, which represented the smallest category at 5.6%. In view of the wide spectrum of wound contamination inherent in the cases, an additional classification along the spectrum of wound contamination, distributing cases among clean/clean-contaminated and contaminated/dirty groups, was adopted, permitting refinement of subsequent statistical comparisons along the relevant gradient of risk.

In terms of departmental distribution, the majority of patients (33.3%) underwent surgery in the General Surgery department, followed by the Gynaecology and Obstetrics department (23.3%) and the Urology department (16.7%). Other departments included Vascular Surgery (13.3%), Orthopaedic Surgery (11.1%), and both Cardiac and Ear, Nose and Throat (ENT) departments (1.1% each), as detailed in Tables 3, 4.

**Table 3:** Types of surgical procedures

Surgical procedure	Frequency (n)	Percentage (%)
Cholecystectomy	12	13.30%
Diabetic foot surgery	12	13.30%
Other (thyroidectomy, mastectomy, colectomy, splenectomy, hemorrhoidectomy)	12	13.30%
Cesarean section	10	11.10%
Orthopaedic surgery	10	11.10%
Appendectomy	8	8.90%
Kidney cyst removal	8	8.90%
Urinary stone management	7	7.80%
Hernia repair	6	6.70%
Perineal tear repair	5	5.60%

**Table 4:** Departmental distribution of the procedures

Specialty/Department	Frequency (n)	Percentage (%)
General Surgery	30	33.30%
Gynaecology & Obstetrics	21	23.30%
Urology	15	16.70%
Vascular Surgery	12	13.30%
Orthopaedic Surgery	10	11.10%
Cardiac Surgery	1	1.10%
Ear, Nose and Throat	1	1.10%

Postoperative outcomes

The length of hospital stay varied among patients, with a mean duration of 3.07 ± 2.04 days and a range from one to 12 days. Regarding postoperative antibiotic use, the mean duration of administration was 6.5 ± 2.02 days, ranging from three to 13 days. These outcomes reflect the typical postoperative management of patients with suspected or confirmed SSIs in the study setting and are summarised in Table 5.

**Table 5:** Postoperative length of stay and antibiotic duration (n=90)

Outcome	Mean ± SD	Range
Length of hospital stay (days)	3.07 ± 2.04	1-12
Duration of postoperative antibiotics (days)	6.5 ± 2.02	3-13

Culture results and distribution of isolated pathogens

Microbiological culture results revealed that *Staphylococcus aureus* was the most frequently isolated pathogen, accounting for 31.1% of positive cultures. *Escherichia coli *and coagulase-negative staphylococci (CoNS) each represented 16.7% of isolates. Other organisms included *Klebsiella* spp. (14.4%), *Pseudomonas aeruginosa* (7.8%), *Proteu*s spp. (4.4%), *Streptococcus* spp. (3.3%), and *Candida* spp. (3.3%). Less common isolates such as *Serratia* and *Enterobacter* were identified in one case each (1.1%). These findings are detailed in Table 6.

**Table 6:** Distribution of microbial isolates from surgical site infections (n=90)

Microorganism	Frequency (n)	Percentage (%)
Staphylococcus aureus	28	31.1%
E. coli	15	16.7%
Coagulase-negative staph (CoNS)	15	16.7%
Klebsiella spp.	13	14.4%
Pseudomonas aeruginosa	7	7.8%
Proteus spp.	4	4.4%
Streptococcus spp.	3	3.3%
Candida spp.	3	3.3%
Serratia spp.	1	1.1%
Enterobacter spp.	1	1.1%

Clinical presentation of SSIs

The most frequently observed clinical sign among patients diagnosed with SSIs was localized redness at the wound site, reported in 96.7% of cases. Pain was also a common symptom, affecting 76.7% of patients, followed by purulent discharge in 64.4% of cases. Fever was the least frequent symptom, present in 42.2% of patients. These findings emphasize the importance of local wound assessment in the early detection of SSIs, as outlined in Table 7.

**Table 7:** Distribution of clinical signs and symptoms of surgical site infections (n=90)

Sign/Symptom	Frequency (n)	Percentage (%)
Redness	87	96.7%
Pain	69	76.7%
Discharge (Purulence)	58	64.4%
Fever (>38°C)	38	42.2%

Laboratory findings

Laboratory analysis revealed a mean haemoglobin level of 10.2 ± 1.1 g/dL, indicating mild anaemia in a significant portion of patients. Serum albumin levels averaged 4.05 ± 0.61 g/dL, while mean white blood cell count (total leukocyte count or TLC) was elevated at 14.1 ± 2.4 ×10³/μL, suggesting an ongoing inflammatory or infectious process. Liver enzyme levels remained within the upper normal range, with alanine aminotransferase (ALT) at 29.3 ± 6.3 IU/L and aspartate transaminase (AST) at 27.9 ± 6.3 IU/L. The full laboratory profile is presented in Table 8.

**Table 8:** Laboratory parameters of patients with SSIs (n=90) SSI: Surgical site infections; ALT: Alanine aminotransferase; AST: Aspartate transaminase

Laboratory parameter	Mean ± SD	Range
Haemoglobin (g/dL)	10.2 ± 1.1	8-13
Albumin (g/dL)	4.05 ± 0.61	3- 5.5
Total leukocyte count (×10³/μL)	14.1 ± 2.4	10-21
ALT (IU/L)	29.3 ± 6.3	9-38
AST (IU/L)	27.9 ± 6.3	8-38

Comparison of antibiotic sensitivity testing methods

A comparison between the results of the Kirby-Bauer disk diffusion method and the automated VITEK-2 system revealed a high level of agreement across multiple antibiotics. For example, both methods showed identical resistance rates for ciprofloxacin and ampicillin, with a Kappa coefficient of 1.00 (p<0.001), indicating perfect concordance. Similarly, strong agreement was observed for ampicillin-sulbactam (κ=0.93), ceftazidime (κ=0.88), moxifloxacin (κ=0.87), and erythromycin (κ=0.86). The lowest level of agreement was recorded with gentamicin (κ=0.51), although it remained statistically significant. These findings demonstrate the reliability of both methods, while highlighting the added value of automated systems in confirming resistance profiles. Detailed comparisons are presented in Table 9.

**Table 9:** Agreement between disk diffusion and VITEK-2 antibiotic sensitivity results VITEK-2 system (bioMérieux, France); Kirby-Bauer disk diffusion method (Thermo Fisher Scientific, Basingstoke, UK)

Antibiotic	Kappa Coefficient (κ)	p-value	Test used	Interpretation
Ciprofloxacin	1	< 0.001	Cohen’s Kappa	Perfect agreement
Ampicillin	1	< 0.001	Cohen’s Kappa	Perfect agreement
Ampicillin-Sulbactam	0.93	< 0.001	Cohen’s Kappa	Almost perfect
Ceftazidime	0.88	< 0.001	Cohen’s Kappa	Strong agreement
Moxifloxacin	0.87	< 0.001	Cohen’s Kappa	Strong agreement
Erythromycin	0.86	< 0.001	Cohen’s Kappa	Strong agreement
Meropenem	0.78	< 0.001	Cohen’s Kappa	Substantial agreement
Vancomycin	0.74	< 0.001	Cohen’s Kappa	Substantial agreement
Cefuroxime	0.71	< 0.001	Cohen’s Kappa	Substantial agreement
Clindamycin	0.64	< 0.001	Cohen’s Kappa	Moderate agreement
Linezolid	0.59	< 0.001	Cohen’s Kappa	Moderate agreement
Amikacin	0.56	< 0.001	Cohen’s Kappa	Moderate agreement
Gentamicin	0.51	< 0.001	Cohen’s Kappa	Moderate agreement
Levofloxacin	0.47	< 0.001	Cohen’s Kappa	Fair agreement

Impact of diabetes mellitus on SSI-related variables

Subgroup analysis comparing patients with and without diabetes revealed a statistically significant association between diabetes and both the department of admission and the length of hospital stay. Diabetic patients were more frequently admitted to the vascular surgery department (26.1%) compared to none among the non-diabetic group (p<0.05). Additionally, the average length of hospital stay was significantly longer for patients with diabetes (3.6 ± 2.08 days) compared to those without diabetes (2.6 ± 1.9 days). However, no statistically significant differences were found between diabetic and non-diabetic groups regarding culture results, clinical signs of SSI, or duration of postoperative antibiotic therapy. These findings are summarized in Table 10.

**Table 10:** Comparison between the patients with SSIs and with or without diabetes SSI: Surgical site infections; ^*^statistical significance (p<0.05)

Variable	Patients with diabetes (n=46)	Patients without diabetes (n = 44)	Test used	Test statistic	p-value
Admission to vascular surgery department	26.10%	0%	Chi-square test	χ² = 12.47	0.0004*
Length of hospital stay (days)	3.6 ± 2.08	2.6 ± 1.9	Mann-Whitney U	U = 680.5	0.031*
Positive culture rate	No difference	No difference	Chi-square test	χ² = 0.41	0.52
Signs & symptoms of SSI	No difference	No difference	Chi-square test	χ² = 0.78	0.38
Duration of antibiotics (days)	No difference	No difference	Mann-Whitney U	U = 910.0	0.19

## Discussion

Postoperative wound infections are defined as infections that develop within 30 days after surgery or within one year in cases involving implants, provided the infection is linked to the surgical procedure. These infections can arise from multiple sources, with hospital-acquired or nosocomial infections being the most common. Their occurrence varies depending on the healthcare setting [8]. 

The investigation revealed that *Staphylococcus aureus* ranked as the predominant microorganism identified in SSIs, followed by *Escherichia coli* and CoNS. The isolates exhibited elevated resistance proportions to ampicillin and gentamicin, but ciprofloxacin and meropenem preserved comparatively stronger antibacterial efficacy. The agreement between disc diffusion and VITEK-2 susceptibility methods was found to be outstanding. Furthermore, diabetic individuals manifested statistically extended lengths of hospitalisation and a markedly higher probability of referral to vascular surgical units 

The findings revealed that the mean age of participants was 42.3 years (ranging from seven to 90 years), with two-thirds being female (66.7%). Over half of the patients were obese (53.3%), approximately one-quarter were smokers (24.4%), around half had diabetes (51.1%), and 35.6% were hypertensive. Additionally, 80% had coexisting comorbidities. These results align with those of Shakir et al. [8], who reported a mean age of 29.9 ± 10.61 years among patients with SSIs, with 59.2% being female and 15.69% smokers. In that study, 33.3% of SSI patients had diabetes. Most patients received antimicrobial prophylaxis (91%) and 80.6% had drains placed at the surgical site. Similarly, Pateriya et al. [9] noted risk factors for SSIs, including diabetes mellitus (13.58%), hypertension (27.6%), anemia (24.69%), and smoking (35.80%) 

The current study demonstrated that the most common bacterial isolate was *Staphylococcus aureus*, consistent with the reports of Mukagendaneza et al. [10] in Rwanda and Ennab et al. [11] in Jordan. In contrast, Pateriya et al. [8] from India reported *Escherichia coli *(30.9%) as the most common pathogen, followed by* Klebsiella pneumoniae *(23.4%) and *Staphylococcus aureus *(14.9%). These differences across geographic locations may reflect variations in surgical practices, infection control measures, and patient populations 

Our findings regarding antimicrobial resistance patterns revealed high resistance rates to ampicillin and gentamicin, while ciprofloxacin and meropenem retained relatively better activity. Similar resistance patterns were documented by Shakir et al. [8] in Ethiopia and Ennab et al. [11] in Jordan. These similarities highlight a broader regional concern regarding the misuse of antibiotics and the urgent need for antibiotic stewardship programs 

The high level of agreement observed between the Kirby-Bauer disk diffusion method and the automated VITEK-2 system in our study underlines the reliability of both techniques. Similar concordance was reported by Strauss et al. [12] and Rampacci et al. [13], who compared conventional methods with automated systems and found strong levels of agreement. This supports the use of VITEK-2 as a confirmatory tool in resource-limited settings where rapid and accurate results are needed 

The association between diabetes mellitus and prolonged hospital stay, as well as the higher likelihood of referral to vascular surgical units observed in our study, is consistent with findings from Al-Awaysheh et al. [14]. Their work highlighted diabetes as a major risk factor for postoperative complications and extended recovery times, reinforcing the need for stringent perioperative glycemic control. 

The variability in the distribution of microorganisms across different studies highlights the influence of geographical location, infection control practices, and patient-related risk factors. For instance, while *S. aureus* predominated in our setting as well as in Mukagendaneza et al. [10] and Shakir et al. [8], Gram-negative organisms such as *E. coli* and *Klebsiella* spp. were more frequent in other reports [9,15]. These differences emphasize the importance of local surveillance data in guiding empirical antibiotic therapy. 

Forensic implications 

While not the principal concern of the current investigation, SSIs possess distinct forensic and medico-legal significance. The forensic inquiry ultimately pivots on the classification of the infection; it must be determined whether the event qualifies as an inevitable surgical complication or whether it denotes non-compliance with established perioperative protocols. The observed prevalence of multidrug-resistant bacteria and the protracted hospitalisation of the cohort with diabetes underscore the critical necessity for unwavering adherence to infection prevention measures and prompt clinical response. Deviations in sterile technique, insufficient monitoring of postoperative wounds, or procrastinated antibiotic therapy can all be construed as lapses that invite allegations of negligence in tort or regulatory proceedings [16-18]. 

Further complicating the medico-legal evaluation is the question of documentation. Gaps in operative reporting or inadequate chronicle of the wound’s postoperative trajectory may undermine a surgeon’s ability to mount an effective defence in litigation or disciplinary review. In contrast, meticulous records that chronicle the informed consent process, the administration of prophylactic antimicrobials, and the regimen of wound management together furnish compelling evidence of adherence to the established standard. Previous forensic discourse attests to the dual protective function of precise documentation: it safeguards patient safety while simultaneously fortifying the legal position of surgical practitioners [2,19,20]. 

Consequently, in addition to their microbiological and clinical ramifications, SSIs underscore the surgical team’s bifurcated ethical and professional imperative: to sustain uncompromising standards of infection prophylaxis and to chronicle every facet of perioperative stewardship. Adherence to these protocols diminishes patient morbidity while concurrently curtailing the spectrum of legal exposure, thereby illuminating the inseparability of clinical efficacy and medico-legal responsibility. 

Limitations

The present investigation is subject to a number of methodological constraints. Primarily, recall bias may have shaped the accuracy of self-reported information on preoperative interventions, including the administration of prophylactic antibiotics and the chosen shaving technique. Additionally, selection bias cannot be entirely discounted, given the deliberate exclusion of patients with poorly controlled diabetes and certain other systemic diseases; this may have led to an underestimation of the actual incidence of surgical site infections. Furthermore, the follow-up period was confined to 90 days after surgery, thereby limiting the capacity to identify infections that manifest later or to evaluate outcomes beyond this horizon. To robustly test and elaborate upon these observations, future multicentric investigations should enroll more extensive patient cohorts and adopt a prolonged follow-up paradigm. 

## Conclusions

This investigation, conducted at a single institution, reinforces that SSIs constitute a persistent clinical concern, driven notably by multidrug-resistant *Staphylococcus aureus* and extended-spectrum beta-lactamase-producing *E. coli*. The data advocate for the immediate adoption of localized infection control measures, emphasizing the tightening of perioperative antibiotic protocols and the stratified surveillance of vulnerable cohorts, particularly patients with diabetes mellitus. The striking burden of resistance also supports the strategic application of tailored antibiotic prophylaxis and the possible incorporation of MRSA screening among discrete surgical populations. Collectively, these tailored interventions may refine postoperative outcomes and simultaneously guide system-wide infection prevention directives in analogous resource-constrained environments.
